# Deep learning radiomics model based on breast ultrasound video to predict HER2 expression status

**DOI:** 10.3389/fendo.2023.1144812

**Published:** 2023-04-18

**Authors:** Meng-Yao Quan, Yun-Xia Huang, Chang-Yan Wang, Qi Zhang, Cai Chang, Shi-Chong Zhou

**Affiliations:** ^1^ Department of Ultrasonography, Fudan University Shanghai Cancer Center, Shanghai, China; ^2^ Department of Oncology, Shanghai Medical College, Fudan University, Shanghai, China; ^3^ Laboratory of The Smart Medicine and AI-based Radiology Technology (SMART), School of Communication and Information Engineering, Shanghai University, Shanghai, China

**Keywords:** ultrasound, breast cancer, human epidermal growth factor receptor 2, deep learning, YOLO V5, radiomics

## Abstract

**Purpose:**

The detection of human epidermal growth factor receptor 2 (HER2) expression status is essential to determining the chemotherapy regimen for breast cancer patients and to improving their prognosis. We developed a deep learning radiomics (DLR) model combining time-frequency domain features of ultrasound (US) video of breast lesions with clinical parameters for predicting HER2 expression status.

**Patients and Methods:**

Data for this research was obtained from 807 breast cancer patients who visited from February 2019 to July 2020. Ultimately, 445 patients were included in the study. Pre-operative breast ultrasound examination videos were collected and split into a training set and a test set. Building a training set of DLR models combining time-frequency domain features and clinical features of ultrasound video of breast lesions based on the training set data to predict HER2 expression status. Test the performance of the model using test set data. The final models integrated with different classifiers are compared, and the best performing model is finally selected.

**Results:**

The best diagnostic performance in predicting HER2 expression status is provided by an Extreme Gradient Boosting (XGBoost)-based time-frequency domain feature classifier combined with a logistic regression (LR)-based clinical parameter classifier of clinical parameters combined DLR, particularly with a high specificity of 0.917. The area under the receiver operating characteristic curve (AUC) for the test cohort was 0.810.

**Conclusion:**

Our study provides a non-invasive imaging biomarker to predict HER2 expression status in breast cancer patients.

## 1 Introduction

Global cancer statistics for 2020 show that breast cancer has the highest incidence of all cancers in women and is the second leading cause of tumor-related death (1). HER2 is a transmembrane tyrosine kinase receptor whose gene is located on human chromosome 17q21 (2, 3). HER2 overexpression and gene amplification, which account for 15-20% of breast cancer patients, are important prognostic factors in breast cancer and influence the choice of therapeutic agents for breast cancer patients (4). Prior to the introduction of HER2-targeted drugs such as trastuzumab and patolizumab, HER2-positive patients had high recurrence rates and poor survival outcomes (3, 5). Trastuzumab considerably increased progression-free survival and overall survival in patients with early and advanced HER2-positive breast cancer, according to landmark trial data till 2006 (5–7).. An accurate evaluation of HER2 status is essential in the treatment of breast cancer since HER2-targeted therapy is only effective in tumors with HER2 overexpression and/or gene amplification (7–9).. Currently, *in situ* hybridization to assess HER2 gene amplification and immunohistochemistry (IHC) to assess protein overexpression are still the primary methods to detect HER2 expression status (10, 11). However, heterogeneity in intra-tumor HER2 expression has been reported by several research institutions, with an incidence of nearly 40% (12, 13). Preoperative core needle biopsy (CNB) can only obtain tissue from a portion of the lesion and cannot assess HER2 expression in the entire lesion. It has been shown that the concordance rate of HER2 expression status between preoperative CNB and subsequent histopathology of the resected lesion ranges from 81% to 97% (14–16). Furthermore, 20-40% of patients who receive neoadjuvant chemotherapy have altered HER2 expression (17, 18). And it has been demonstrated that HER2 intra-tumoral heterogeneity is an independent factor linked to HER2-positive patients’ insufficient response to neoadjuvant chemotherapy (19). Therefore, there is an urgent need for real-time detection of changes in HER2 expression status during neoadjuvant chemotherapy. However, in clinical practice, it is difficult for us to assess tumor HER2 expression status in real time through multiple pre-operative multi-site CNB, and pre-operative CNB are difficult to circumvent the potential bias caused by intra-tumoral heterogeneity of HER2.Therefore, it is difficult to achieve clinical real-time detection and evaluation and individualized treatment. In summary, we need to find an accurate, convenient, and non-invasive method to predict HER2 expression status to guide the individualized treatment of breast cancer patients and improve their prognosis.

Ultrasound is one of the routine preoperative examinations for breast cancer and has been widely used for the preoperative characterization of breast lesions because it is non-invasive and easy to perform. The molecular staging of breast cancer has been shown to correlate with its ultrasound characteristics (20–22). Radiomics has been widely used in the diagnosis of breast cancer (23). Although the term is not strictly defined, radiomics usually aims to extract a large amount of image information from ultrasound, CT, and other imaging images in a high-throughput manner to realize tumor segmentation, feature extraction, and model building. These features are often difficult to identify by the human eye and can be deeply correlated with the intrinsic qualities of the lesion through quantification, so radiology performs better than traditional imaging methods (24). However, the analysis of ultrasound images by radiomics still has some limitations, such as the need to manually depict the ROI to achieve tumor segmentation, which may affect the extraction of feature values (25, 26). Convolutional neural networks have had tremendous success in bioinformatics since 2012 thanks to the development of deep learning, particularly in medical imaging (27). A recent study shows that DLR can be used to analyze US images for prediction of HER2 expression status (28). However, DLR usually faces the problem of small sample learning, and this study had only 36 patients in the validation set and 108 patients in the training set. In addition, the sensitivity and specificity of the prediction model built in this study were not high, at 72.73% and 84.00%, respectively. In the process of extracting image features by DLR, it is common practice to require the sonographer to record one or several representative frames of the lesion during the examination and to perform feature extraction based on this. However, existing computer-aided classification tools tend to focus only on the final classification results and ignore the impact of key frame selection. The challenge of identifying features associated with lesions persists, along with a significant category imbalance (29).

Clinical parameters combined DLR, which integrates clinical data with network features, assists in giving information that is complementary to image features and builds models by utilizing clinical data and US image features in concert, enhancing model performance (30). It has been proposed that clinical T-stage, N-stage, and age may correlate with HER2 expression status (31). Therefore, in order to comprehensively assess HER2 expression status and improve the diagnostic performance of ultrasound prediction of HER2 expression status, we developed a clinical parameters combined DLR model based on the YOLO v5 deep neural network, which combines the time-frequency domain features of breast lesions in breast ultrasound videos with clinical parameters, and achieves both innovative 3D feature extraction of breast lesions and accurate real-time assessment of HER2 expression status.

## 2 Materials and methods

### 2.1 Patients

Data for this retrospective study were obtained from 807 patients with breast lesions who consulted the ultrasound department of the Fudan University Shanghai Cancer Center from February 2019 to July 2020. The inclusion criteria were as follows: (a) patients who underwent ultrasound within one week before surgery and whose ultrasound images showed suspected breast lesions; (b) clinical data were available; and (c) patients with the intention of undergoing breast cancer surgery. Exclusion criteria were: (a) patients who underwent CNB before ultrasonography; (b) missing primary clinical data or ultrasound video data; (c) non-compliant ultrasound video acquisition; (d) patients with multifocal lesions or bilateral disease; (e) patients with postoperatively pathologically confirmed non-invasive breast cancer and patients with benign lesions; (f) non-lumpy lesions.

### 2.2 Clinical characteristics

Obtained clinical and histopathological data from medical records. Histopathological findings of breast cancer include tumor type and the HER2 proliferation index. Clinical data included the patient’s menopausal status, age, US size (The transverse diameter of the largest cross-section of the lesion in ultrasonography was used as the standard), N stage, T stage, and TNM stage. According to the American Society of Clinical Oncology/College of American Pathologists Clinical Practice Guideline, patients were divided into a HER2 positive group and a HER2 negative group (10). HER2-negativity was defined as IHC 0, IHC 1+, or IHC 2+ and lack of HER2 gene amplification measured by *in situ* hybridization. HER2-positivity was defined as IHC 2+ or 3+ with HER2 gene amplification.

### 2.3 Ultrasound videos collection

Preoperative breast ultrasonography was performed by an ultrasonographer with more than 5 years of experience in breast ultrasonography, using a SuperSonic Imagine S.A, Aix-en-Provence, France color Doppler ultrasound diagnostic instrument with a selected frequency of 7~13MHz linear array transducer. During the examination, the area of the breast lesion is captured in Digital Imaging and Communications in Medicine (DICOM) format, and each frame of the video is extracted and stored in JPG format. The video acquisition method and criteria were as follows: The depth was uniformly set to 3.5 cm (including the subcutaneous fat layer and superficial pectoral muscle layer), the gain was uniformly set to 49%, and the ROI was placed in the middle of the image to obtain a standardized breast ultrasound video. The video was divided into frames for each case on the original video data to facilitate subsequent analysis.

### 2.4 Deep learning radiomics models

**Figure 1:**
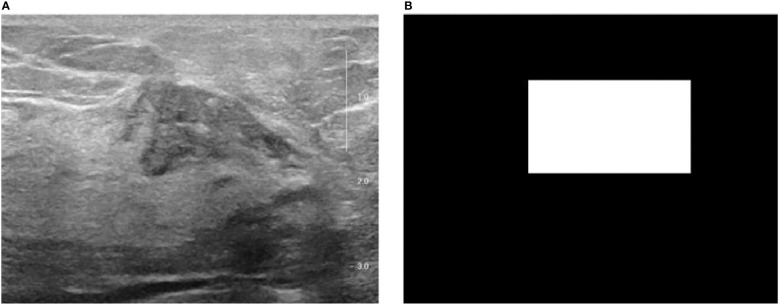
The original breast ultrasound image and their corresponding masked image. **(A)** Original image; **(B)** Masked image.

**Figure 2:**
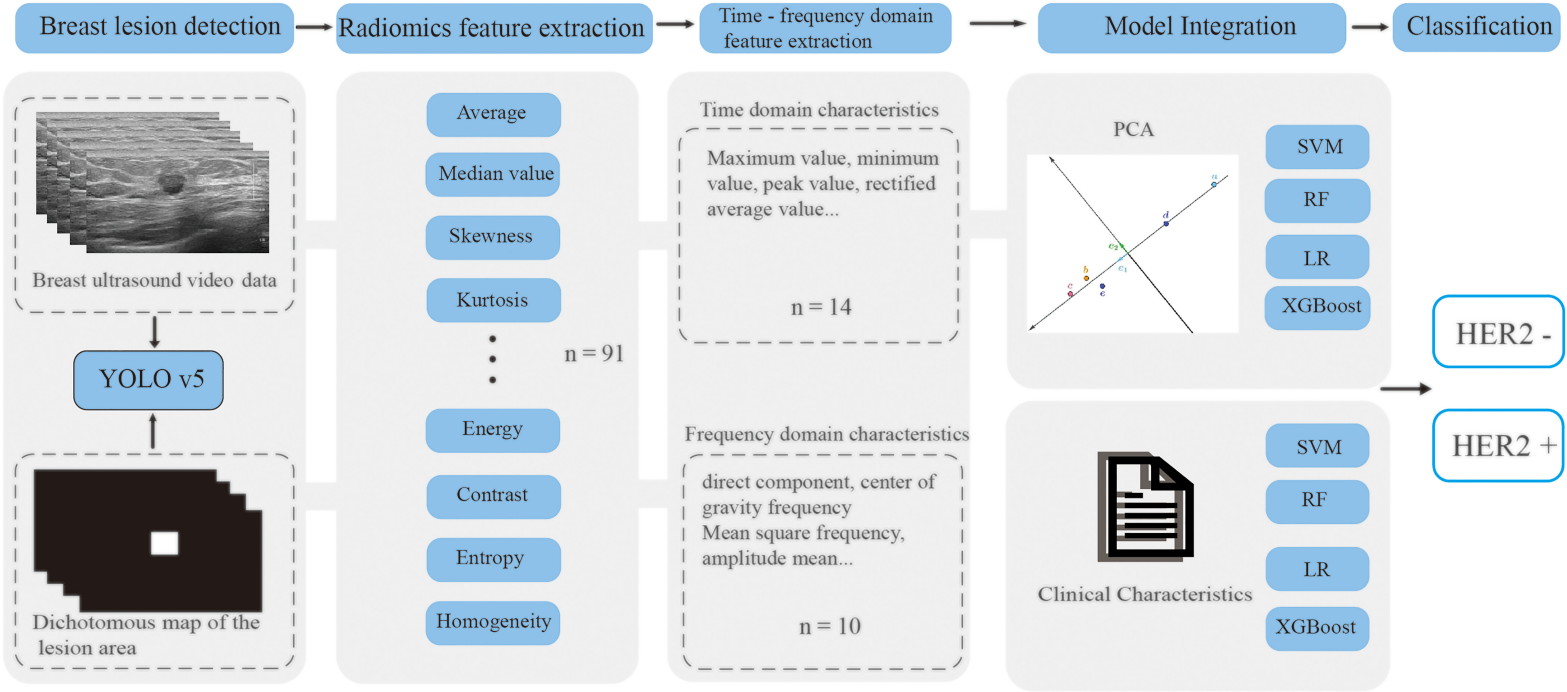
The overall model construction process. PCA, Principal Component Analysis.

The enrolled patients were randomly divided into a training cohort and an independent test cohort in a 4:1 ratio, with the training cohort used to optimize the model parameters and the independent test cohort used for model validation (32). The multi-stage DLR-based HER2 negative-positive discrimination model proposed in this paper consists of the following analytical steps (1): A lesion detection model based on YOLO v5 neural network is trained for breast video sequences to achieve accurate localization of lesions in each frame and generate binary images of lesion regions (**Figure 1**) (2); 91 radiomics features were extracted from each still image frame and its mask image (3); Based on the video sequence, 24 time and frequency domain features are extracted for each static radiomics feature to form the radiomics features of the dynamic video (4); The classifier based on time-frequency domain features is integrated with the classifier of clinical variables, and the integrated model outputs the final HER2 negative-positive discrimination results (**Figure 2**).

#### 2.4.1 Detection of breast lesions

In breast ultrasound video, the size and shape of the tumor and its distribution in the image are generally not fixed, so finding a method to accurately localize the location of the breast lesion is critical for subsequent analysis. This paper trains a breast lesion detection model based on the YOLO v5 deep neural network to achieve automatic and fast localization of lesions in ultrasound videos (33, 34).

#### 2.4.2 Static radiomics feature extraction

The trained YOLO v5 detection model outputs the coordinates of the lesion on each breast ultrasound image frame and ensures the accuracy and coherence of the model’s detection on the overall video through subsequent rejection and interpolation processes. According to each frame of the image in **Figure 1** and its corresponding mask image, its spatial domain features can be extracted for each lesion, including first-order statistical features, binary texture features, and gray-level co-generation matrix features (35).

#### 2.4.3 Dynamic time-frequency domain feature extraction

For the dynamic characteristics of breast ultrasound video, The dynamic change curve of each static feature in time can be constructed, from which a total of 14 time-domain features can be extracted. At the same time, the fast Fourier transform of the time domain signal can obtain the frequency spectrum of the signal, and then extract a total of 10 frequency domain features, such as the direct component (dc), center of gravity frequency (fc), mean square frequency (msf).

### 2.5 Statistical analysis

Clinicopathological differences between the training and test sets were compared by t-test or Mann-Whitney U-test. AUC, accuracy, sensitivity, specificity, and the Youden index were used to evaluate the performance of the HER2 expression status assessment model. The AUC, accuracy, and YI differences between integrated models were compared using the paired t-test. P values less than 0.05 were regarded as statistically significant for all two-sided statistics. Statistical analysis was performed using SPSS and R software.

## 3 Results

### 3.1 Baseline characters

**Figure 3:**
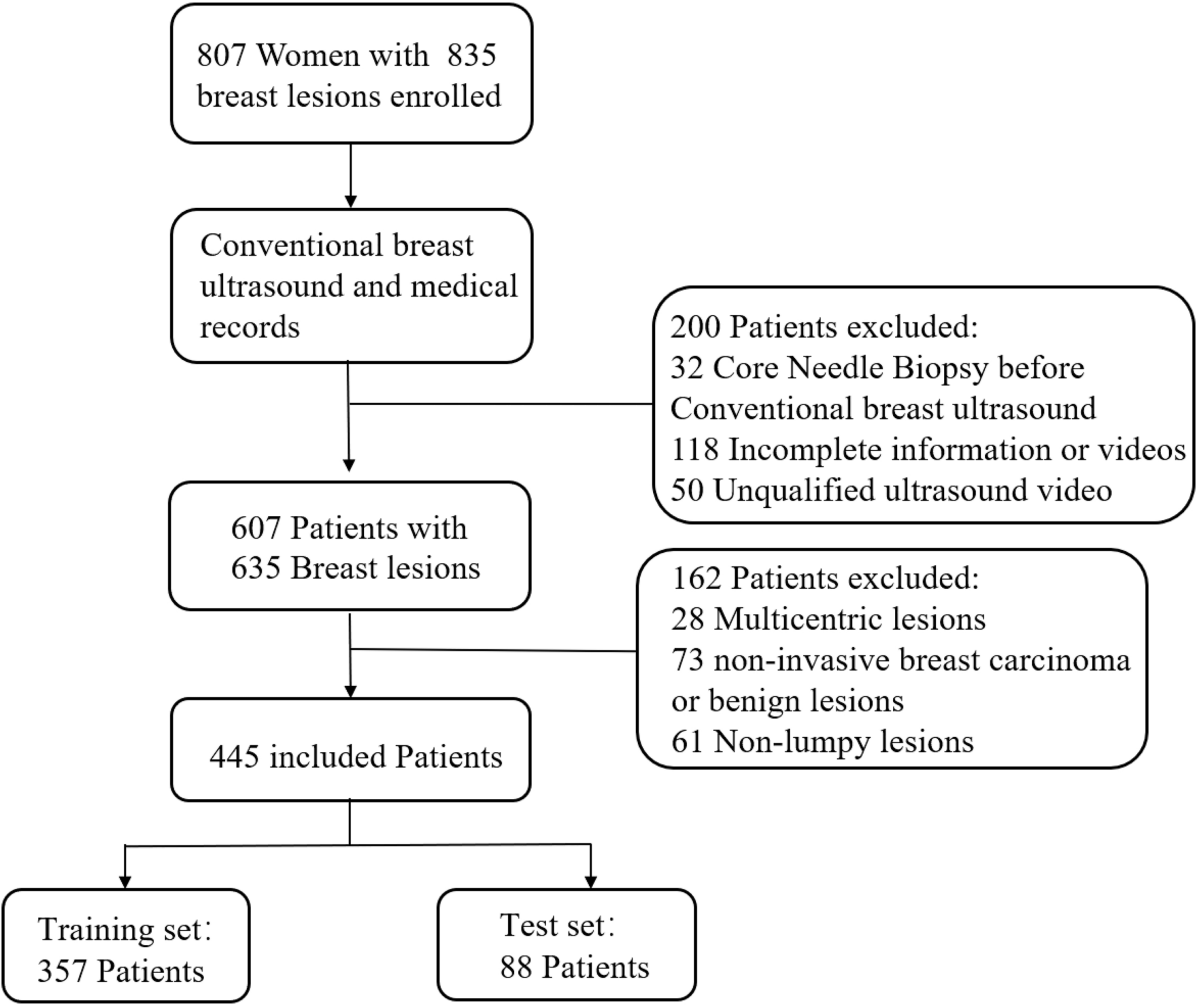
Patient recruitment workflow. In total, 835 out of 807 patients were included according to the selection criteria.

**Figure 4:**
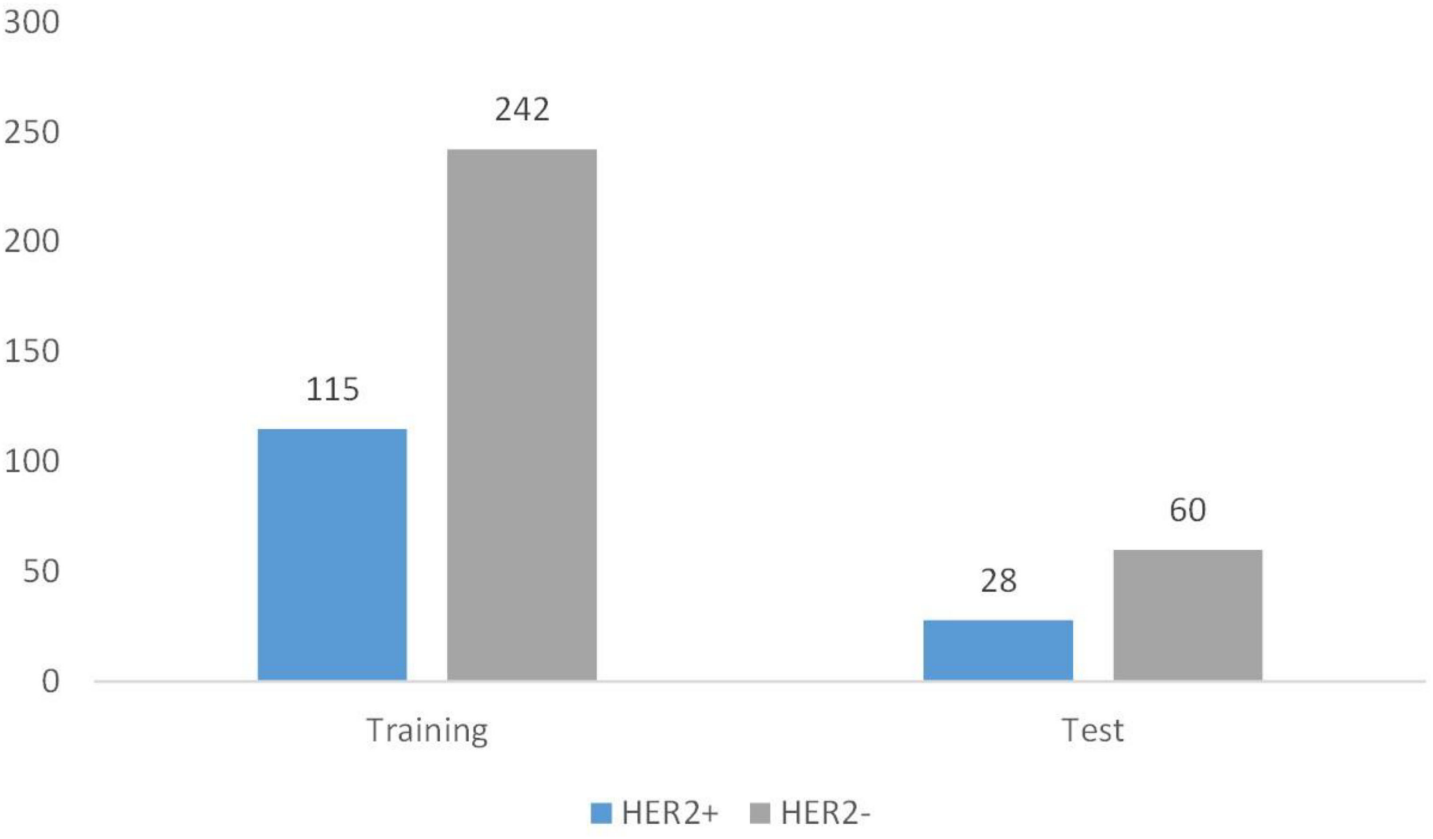
Patient grouping chart. A total of 445 patients. Among them, there are 357 cases in the training set and 88 cases in the test set.

Between January 2019 and April 2020, 807 women with 835 breast lesions were examined; ultimately, 445 women (mean age 50 years; age range 26-83 years) with 445 malignant breast lesions were included in the study. **Figure 3** shows the patient recruitment workflow. The training set contained 357 patients, while the test set contained 88 patients. Menopausal status, age, US size, N stage, T stage, and TNM stage parameters did not significantly differ between the training and test sets (**Table 1**). According to the results of IHC or FISH, 115 patients in the training set were HER2-positive and 242 patients were HER2-negative, and 28 patients in the validation set were HER2-positive and 60 patients were HER2-negative (**Figure 4**).

**Table 1:** Patient and tumor characteristics.

Characteristics	Total	Training	Test	P
Number	445	357 (80%)	88(20%)	
Age	51.87 ± 10.62	52.20 ± 10.67	50.52 ± 10.38	0.185
Size	2.36 ± 1.11	2.37 ± 1.15	2.33 ± 0.98	0.767
Meno				0.593
0	186 (41.8%)	147 (41.2%)	39 (44.3%)	
1	259 (58.2%)	210 (58.8%)	49 (55.7%)	
cN				0.464
0	342 (76.9%)	277 (77.6%)	65 (73.8%)	
1	98 (22.0%)	76 (21.3%)	22 (25%)	
2	5 (1.1%)	4 (1.1%)	1 (1.2%)	
cT				0.942
1	215 (48.3%)	173 (48.4%)	42 (47.7%)	
2	223 (50.1%)	178 (49.9%)	45 (51.1%)	
3	7 (1.6%)	6 (1.7%)	1 (1.2%)	
cTNM				0.895
1	186 (41.8%)	149 (41.7%)	37 (42.0%)	
2	251 (56.4%)	201 (56.3%)	50 (56.8%)	
3	8 (1.8%)	7 (2.0%)	1 (1.2%)	

Qualitative variables are in n (%) and quantitative variables are in mean ± SD, when appropriate.

Meno Menopause conditions.

### 3.2 Base model selection

**Figure 5:**
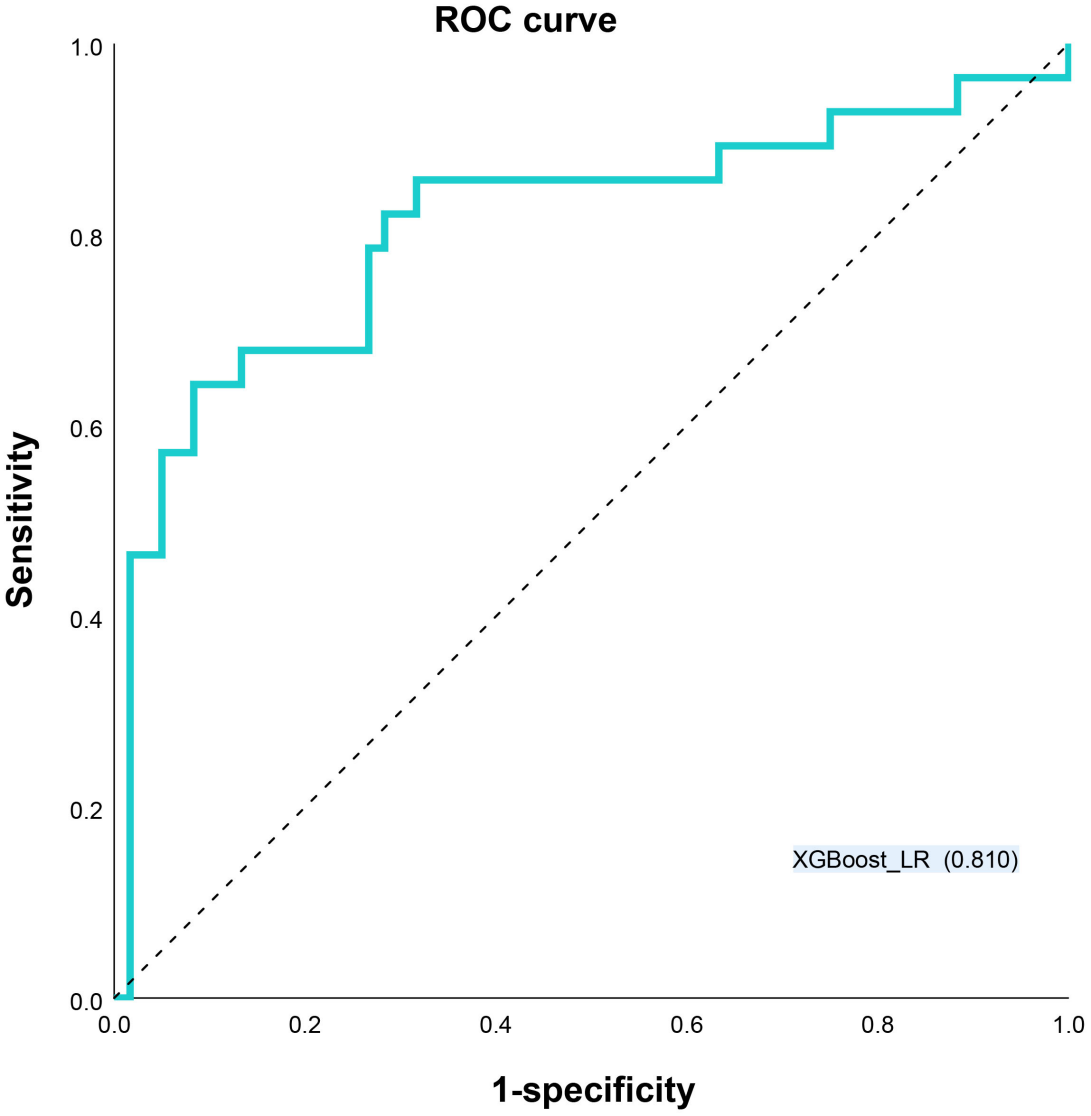
Receiver operating characteristic (ROC) curves XGBoost_LR models for predicting HER2 status. LR Logistic Regression, XGBoost Extreme Gradient Boosting.

**Figure 6:**
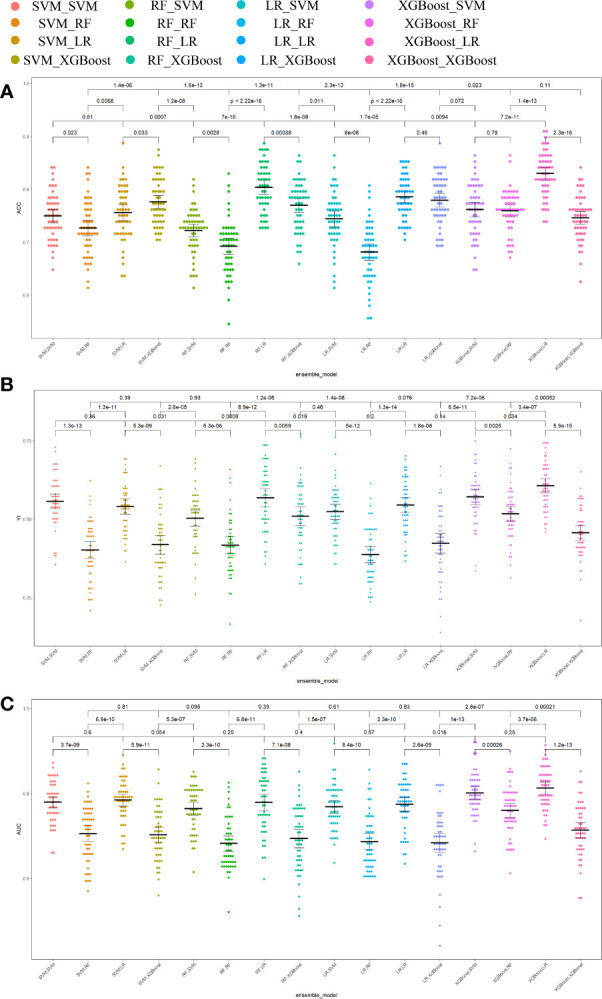
Comparison of prediction performance metrics of each integrated model in 1000-time repeated validation. **(A)** Comparison of the Youden Index of the models. **(B)** Comparison of the accuracy of the models. **(C)** Comparison of the AUCs of the models. ACC accuracy, YI Youden index, AUC area under the receiver operating characteristic curve.

Different base models act as classifiers and will have a significant impact on classification. To figure out the ideal base model for the HER2 prediction task, we compared the performance of Support Vector Machine (SVM), Random Forest (RF), LR and XGBoost in predicting HER2 expression status. First, the classifiers based on time-frequency domain features are constructed, and their specific statistics are shown in **Table 2**. The specificity of all four classifiers based on time-frequency domain features is excellent, and the classification model with RF as the feature encoder has a high specificity of 100% in the test set. Next, a classifier based on clinical variables was constructed, and its detailed statistical results are shown in **Table 3**. Finally, the specific statistical results of integrating the classifier based on time-frequency domain features with the classifier of clinical variables are shown in **Table 4**. The results showed that the prediction performance of the model integrating the time-frequency domain feature classifier and the clinical variable classifier was better than that of the classification model based on time-frequency domain features or clinical variables only. The maximum AUC value (81%) can be obtained when the time-frequency domain feature classification is based on the base model XGBoost and the clinical variables classification is based on the base model LR for model integration (**Figure 5**). At the same time, this model had a high specificity (91.7%). By comparing the AUC, accuracy, and YI of different integrated models through multiple replicate sampling in the test set (88 random samples in the test set and 1000 repetitions of this process to avoid differences in evaluation results due to data bias), the results all indicate that this base model combined pattern has the best performance in predicting HER2 expression status (**Figure 6**).

**Table 2:** Classification results based on time-frequency domain features.

Classifiers		AUC (%)	ACC (%)	SEN (%)	SPC (%)	YI (%)
SVM	T	95.8 [93.2, 97.8]	94.1 [91.6, 96.6]	87.8 [82.6, 95.3]	97.1 [88.3, 99.2]	84.9 [78.6, 91.3]
	I-T	68.9 [54.9, 80.6]	75.0 [65.9, 84.1]	57.1 [36.8, 90.9]	83.3 [38.1, 96.7]	40.5 [21.2, 60.9]
RF	T	99.3 [98.5, 99.9]	97.5 [95.8, 98.9]	97.4 [93.3, 100.0]	97.5 [96.4, 100.0]	94.9 [91.6, 98.7]
	I-T	65.8 [49.8, 78.7]	80.7 [71.6, 88.6]	39.3 [22.6, 70.8]	100.0 [80.4, 100.0]	39.3 [21.7, 59.1]
LR	T	92.7 [90.0, 95.1]	85.4 [81.8, 88.8]	85.2 [75.4, 95.2]	85.5 [74.3, 93.7]	70.8 [64.6, 79.1]
	I-T	68.2 [54.1, 80.2]	76.2[67.0, 84.1]	50.0 [30.0, 84.6]	88.3 [51.4, 100.0]	38.3 [21.0, 59.8]
XGBoost	T	99.9 [99.7, 100.0]	99.4 [98.6, 100.0]	99.1 [96.9, 100.0]	99.6 [98.7, 100.0]	98.7 [96.5, 100.0]
	I-T	71.6 [56.6, 83.4]	79.5 [71.6, 87.5]	67.9 [46.4, 84.2]	85.0 [77.3, 95.3]	52.9 [33.9, 71.3]

95% conﬁdence intervals are included in brackets. AUC area under the receiver operating characteristic curve, ACC accuracy, SEN sensitivity, SPC speciﬁcity, YI Youden index, LR Logistic Regression, RF Random Forest, SVM Support Vector Machine, XGBoost Extreme Gradient Boosting, T training cohort (n = 357), I–T independent test cohort (n = 88).

**Table 3:** Classification results based on clinical characteristics.

Classifiers		AUC (%)	ACC (%)	SEN (%)	SPC (%)	YI(%)
SVM	T	64.7 [58.5, 70.8]	64.7 [59.4, 69.5]	55.7 [36.5, 85.8]	69.0 [36.5, 85.9]	24.7 [16.1, 35.4]
	I-T	67.1 [53.8, 78.2]	70.5 [60.2, 79.5]	64.3 [42.9, 100.0]	73.3 [27.0, 90.2]	37.6 [20.3,58.5]
RF	T	64.1 [58.6, 69.8]	57.7 [52.7, 62.7]	69.6 [37.0, 77.7]	52.1 [47.8, 84.3]	21.6 [14.6, 33.2]
	I-T	54.8 [41.2, 71.1]	69.3 [60.2, 79.5]	39.3 [22.2, 86.2]	83.3 [35.6, 95.1]	22.6 [7.6, 46.8]
LR	T	63.5 [56.9, 69.6]	64.4 [59.7, 69.5]	56.5 [39.8, 79.0]	68.2 [41.6, 82.9]	24.7 [16.2, 36.7]
	I-T	68.9 [56.5, 79.7]	58.0 [46.6, 67.0]	89.3 [41.7, 100.0]	43.3 [36.34, 90.8]	32.6 [20.3, 56.1]
XGBoost	T	69.0 [62.9, 75.2]	61.9 [56.6, 67.2]	73.9 [52.7, 84.7]	56.2 [47.0, 75.3]	30.1 [21.3, 41.8]
	I-T	61.5 [46.8, 73.9]	61.4 [51.1, 70.5]	64.3 [20.8, 90.6]	60.0 [30.5, 96.6]	24.3 [9.7, 46.2]

95% conﬁdence intervals are included in brackets. AUC area under the receiver operating characteristic curve, ACC accuracy, SEN sensitivity, SPC specificity, YI Youden index, LR Logistic Regression, RF Random Forest, SVM Support Vector Machine, XGBoost Extreme Gradient Boosting, T training cohort (n = 357), I–T independent test cohort (n = 88).

**Table 4:** Classification results of integrated time frequency domain feature and clinical characteristics classification model.

Time-frequency domain characteristics model	Clinical characteristics model		AUC(%)	ACC(%)	SEN(%)	SPC (%)	YI (%)
SVM	SVM	T	96.4 [94.2, 98.2]	93.5 [91.0, 96.1]	87.8 [83.2, 95.3]	96.3 [90.0, 98.8]	84.1 [78.9, 90.6]
		I-T	77.4[67.0,87.9]	75.0[74.6,75.4]	85.7[72.8,98.7]	70.0[58.4,81.6]	55.7[31.2,80.3]
	RF	T	95.9 [93.5, 98.1]	93.8 [91.0, 96.3]	87.8 [83.2, 96.4]	96.7 [89.5, 98.8]	84.5 [79.0, 91.4]
		I-T	71.5[59.6,83.3]	72.7[72.3,73.2]	50.0[31.5,68.5]	83.3[73.9,92.8]	33.3[5.4,61.3]
	LR	T	95.9 [93.5, 98.1]	93.8 [91.0, 96.4]	87.8 [83.2, 96.4]	96.7 [89.5, 98.8]	84.5 [79.0, 91.4]
		I-T	79.0[68.2,89.8]	76.1[75.5,76.5]	75.0[59.0,91.0]	76.7[66.0,87.4]	51.7[25.0,78.4]
	XGBoost	T	96.3 [94.0, 98.2]	94.1 [91.6, 96.4]	87.8 [83.2, 95.5]	97.1 [90.0, 99.2]	84.9 [79.3, 91.5]
		I-T	70.1[57.8,82.4]	77.3[76.9,77.7]	46.4[28.0,64.9]	91.7[84.7,98.7]	38.1[12.7,63.6]
RF	SVM	T	99.4 [98.6, 99.9]	98.0 [96.4, 99.4]	95.7 [92.7, 100.0]	99.2 [96.3, 100.0]	94.8 [91.3, 98.6]
		I-T	78.0[67.0,88.9]	72.7[72.3,73.2]	78.6[63.4,93.8]	70.0[58.4,81.6]	48.6[21.8,75.4]
	RF	T	99.4 [98.5, 99.9]	97.5 [95.8, 98.9]	97.4 [93.2, 100.0]	97.5 [96.4, 100.0]	94.9 [91.8, 98.7]
		I-T	69.2[55.9,82.7	69.3[68.8,69.8]	67.9[50.6,85.2]	70.0[58.4,81.6]	37.9[9.0,66.8]
	LR	T	99.4 [98.6, 99.9]	98.0 [96.4, 99.4]	95.7 [93.0, 99.2]	99.2 [96.3, 100.0]	94.8 [91.5, 98.7]
		I-T	78.6[67.0,90.2]	80.7[80.3,81.0]	67.9[50.6,85.2]	86.7[78.1,95.3]	54.6[28.7,80.5]
	XGBoost	T	99.4 [98.7, 99.9]	98.0 [96.6, 99.9]	95.7 [93.2, 99.2]	99.2 [96.2, 100.0]	94.8 [91.6, 98.3]
		I-T	69.9[55.9,83.8]	77.3[76.9,77.7]	67.9[50.6,85.2]	81.7[71.9,91.5]	49.6[22.5,76.7]
LR	SVM	T	92.9 [90.3, 95.5]	86.3 [82.6, 89.6]	82.6 [76.3, 95.9]	88.0 [74.7, 93.5]	70.6 [65.3, 79.6]
		I-T	76.4[65.4,87.4]	73.9[73.4,74.3	75.0[59.0,91.0]	73.3[62.1,84.5]	48.3[21.1,75.5]
	RF	T	92.9 [89.9, 95.2]	85.2 [81.2, 88.8]	86.1 [77.7, 95.8]	84.7 [74.3, 93.5]	70.8 [64.7, 79.2]
		I-T	70.4[58.1,82.7]	70.5[70.0,70.9]	60.7[42.6,78.8]	75.0[64.0,86.0]	35.7[6.6,64.8]
	LR	T	92.9 [90.2, 95.4]	87.4 [84.0, 90.5]	80.9 [76.4, 96.4]	90.5 [74.7, 93.7]	71.4 [65.2, 79.8]
		I-T	78.3[67.1,89.5]	78.4[78.0,78.8]	67.9[50.6,85.2]	83.3[73.9,92.8]	51.2[24.5,78.0]
	XGBoost	T	93.1 [90.3, 95.5]	84.3 [80.7, 88.0]	93.0 [79.6, 96.6]	80.2 [76.7, 92.4]	73.2 [67.2, 80.5]
		I-T	70.1[57.3,82.8]	78.4[78.0,78.8]	50.0[31.5,68.5]	91.7[84.7,98.7]	41.7[16.2,67.2]
XGBoost	SVM	T	99.9 [99.7, 100.0]	99.4 [98.6, 100.0]	99.1 [97.2, 100.0]	99.6 [98.7, 100.0]	98.7 [96.5, 100.0]
		I-T	80.7[70.5,90.9]	76.1[75.7,76.5]	82.1[68.0,96.3]	73.3[62.1,84.5]	55.4[30.1,80.8]
	RF	T	99.9 [99.7, 100.0]	99.4 [98.6, 100.0]	99.1 [97.1, 100.0]	99.6 [98.7, 100.0]	98.7 [96.5, 100.0]
		I-T	76.7[65.1,88.3]	76.1[75.7,76.5]	71.4[54.7,88.2]	78.3[67.9,88.8]	49.7[22.6,77.0]
	LR	T	99.9 [99.7, 100.0]	99.4 [98.6, 100.0]	99.1 [97.2, 100.0]	99.6 [98.7, 100.0]	98.7 [96.6, 100.0]
		I-T	81.0[69.7,92.2]	83.0[82.6,83.3]	64.3[46.5,82.0]	91.7[84.7,98.7]	56.0[31.2,80.7]
	XGBoost	T	99.9 [99.7, 100.0]	99.4 [98.6, 100.0]	99.1 [97.0, 100.0]	99.6 [98.7, 100.0]	98.7 [96.5, 100.0]
		I-T	73.9[61.0,86.8]	76.1[75.7,76.5]	64.3[46.5,82.0]	81.7[71.9,91.5]	46.0[18.4,73.5]

95% conﬁdence intervals are included in brackets. AUC area under the receiver operating characteristic curve, ACC accuracy, SEN sensitivity, SPC speciﬁcity, YI Youden index, LR Logistic Regression, RF Random Forest, SVM Support Vector Machine, XGBoost Extreme Gradient Boosting, T training cohort (n = 357), I–T independent test cohort (n = 88).

## 4 Discussion

For rapid real-time prediction of HER2 expression status in breast cancer patients, we developed and validated a clinical characteristics combined DLR approach based on breast ultrasound video in this study. This method showed better diagnostic performance in distinguishing HER2-negative patients from HER2-positive patients compared to models based on conventional ultrasound images or clinical characteristics only. It is encouraging that our model shows a higher specificity of 0.910 and is able to obtain a lower false-positive rate, thus reducing ineffective anti-HER2 therapy and patient harm from repeat punctures.

Intratumor heterogeneity of HER2 expression levels has been widely reported (12). On the one hand, IHC and FISH are limited by sample acquisition and can only reflect the local HER2 expression level of the tumor; on the other hand, the prevalence of FISH is limited, and this test is not available in some regions due to technical limitations, with a multicenter study showing that the acceptance rate of this test among breast cancer patients is only 6%. And because of significant differences in testing practices, test results from regional laboratories are often inaccurate compared to centralized laboratories (36). In overview, we need new methods for real-time assessment of HER2 expression status. Several investigators have suggested that HER2 expression status can be assessed using HER2 nanobodies in combination with PET-CT. However, this method is more expensive and complex, which makes it difficult to be widely used in clinical practice; furthermore, the uptake of this HER2 nanoantibody in breast *in situ* lesions is wide and does not differ significantly from the uptake concentration in surrounding tissues (37, 38). Other researchers attempted to develop a PET-CT radiomics mechanistic learning model to predict HER2 expression status, however, the results showed that PET-CT was not sufficient to accurately predict HER2 expression status with an AUC of 0.72-0.76 (39). Xu ZL et al. developed a deep learning model to predict HER2 expression in breast cancer from ultrasound images (28). In common with this study, our results also showed a lower overall diagnostic performance for HER2 status assessment using clinical parameters alone, with an AUC of 0.55-0.69. However, differing from that study: Firstly, this study applies the YOLOV5 deep neural network to achieve automatic detection of breast cancer lesions in breast ultrasound videos and build more complete clinical diagnostic software. This method is fast and accurate, with a detection time of 0.021 seconds for a single image, which means that it can be used in clinical practice in real time. In particular, changes in HER2 expression status can be detected in real time during neoadjuvant chemotherapy. At the same time, the deep neural network has better detection for small lesions (40). Secondly, this study introduces a new method - extracting 3D features of breast lesions from breast ultrasound videos using a deep learning method based on YOLO v5. Compared to the traditional method of taking one or a few representative frames of a breast lesion during an ultrasound scan and extracting two-dimensional features of the lesion from them. This approach allows for a more comprehensive analysis of the breast lesion while avoiding the effects of key frame selection. Finally, we combine deep learning methods with machine learning methods, i.e., we use deep learning methods for automatic tumor detection and feature extraction and different basic machine learning methods as classifiers to filter out the best performing classifier combinations. This not only improves the performance of the model but also reduces the concurrent processing bias and overfitting, which helps in pattern recognition and parameter selection (26).

The present study still has some limitations. To begin with, the experimental results of this paper show that the training set results are generally better than the test set, probably due to the use of monocentric data with more parameters and high model complexity. In addition to the use of neural network models in this study, there may be uniqueness in the representation of the hidden units of the sample data, all of which may lead to the risk of overfitting the experiment. However, it is worth noting that although the experimental results of the training set have better performance compared to the test set, the test set results are improving as the training set results keep improving. Therefore, whether there is an overfitting problem in the actual experimental process needs to be further investigated. Later, we will add external datasets, conduct multi-center experimental studies to improve the generalization performance of the model, and do more comprehensive tests. This study provides a feasible method for real-time monitoring of changes in HER2 expression status during neoadjuvant chemotherapy or targeted therapy. However, more clinical treatment data is still needed to support future applications in the clinical treatment process. Secondly, in this paper, although we have a high specificity, the sensitivity is only 64.3%. However, the sensitivity of our model has been improved compared with the prediction model based on clinical characteristics only. The sensitivity of prediction models for molecular subtypes of breast cancer based on deep learning methods in published articles ranges from 44% to 96%, and our result is within this reasonable range (41). At present, the resolution of ultrasound video is not as good as that of static 2D ultrasound images, and with future advances in imaging technology, this model is expected to achieve better diagnostic performance. Finally, in recent years, a large number of studies have found that patients with low HER2 expression (IHC 1+ or IHC 2+/*in situ* hybridization -) can benefit from antibody-drug conjugates (42–44). Although the development of a trichotomous predictive model of HER2 expression status is an interesting attempt, there is no radiomics model that classifies HER2 expression status more finely.

## 5 Conclusion

A non-invasive and practical technique for assessing HER2 expression status is provided by clinical parameters combined DLR on traditional US videos of the breast. This technique is able to identify the most suitable pharmacological therapies for breast cancer patients. Prospective multicenter validation is expected to yield additional evidence for clinical use in subsequent studies.

## Data availability statement

The raw data supporting the conclusions of this article will be made available by the authors, without undue reservation.

## Ethics statement

The studies involving human participants were reviewed and approved by the Ethics Committee of Cancer Hospital of Fudan University. The patients/participants provided their written informed consent to participate in this study.

## Author contributions

M-YQ, Y-XH, S-CZ, and QZ designed this study. M-YQ, Y-XH, S-CZ, C-YW and QZ conducted the experiment and interpreted the data. M-YQ, C-YW, and Y-XH analyzed the data. All authors approved the final version of this manuscript.
